# AMPA Receptors Exist in Tunable Mobile and Immobile Synaptic Fractions *In Vivo*

**DOI:** 10.1523/ENEURO.0267-21.2021

**Published:** 2021-07-21

**Authors:** Daniel T. Gray, Lindsay M. De Biase

**Affiliations:** 1 Division of Neural System, Memory and Aging, University of Arizona, Tucson, AZ 85724; 2 Evelyn F. McKnight Brain Institute, University of Arizona, Tucson, AZ 85724; 3 Department of Physiology, David Geffen School of Medicine at University of California Los Angeles, Los Angeles, CA 90095

## Abstract

**Highlighted Research Paper:** AMPA Receptors Exist in Tunable Mobile and Immobile Synaptic Fractions *In Vivo*, by Haiwen Chen, Richard H. Roth, Elena Lopez-Ortega, Han L. Tan, and Richard L. Huganir.

Information processing in the nervous system emerges from complex temporal and spatial interactions between fast excitatory synaptic neurotransmission and modulatory signals from other neurochemicals. A critical property of excitatory synapses is that they undergo bidirectional changes in synapse strength in response to experience to encode relevant information for future recall. This process is referred to as Hebbian plasticity (Magee and Grienberger, 2020) and is a critical mechanism by which brain circuits optimize their function for an organism’s unique needs. While it is difficult to overstate the importance of Hebbian plasticity for learning, memory, and behavior, problems arise if this process proceeds unchecked. Excessive synapse strengthening can lead to aberrant excitability with detrimental consequences for cellular health. One of the most intuitive examples of a pathologic condition associated with increased excitability in the nervous system is epileptic activity, and numerous other developmental and neurodegenerative disorders are also associated with excess neuronal activity. Consequently, neuronal networks must be scaled to a healthy physiological state while differences in synapse weightings that arise through Hebbian plasticity are retained. Remarkably, a second form of plasticity that globally scales synapse strength to levels of healthy excitability has evolved in response to these demands (Turrigiano, 2012). This form of plasticity is known as homeostatic plasticity.

Homeostatic plasticity is primarily accomplished through altering the number and composition of excitatory neurotransmitter receptors found at postsynaptic sites (Diering and Huganir, 2018). Glutamate-activated AMPA receptors are the central mediators of fast excitatory neurotransmission in the central nervous system. It has been known for some time that AMPA receptors can diffuse along a neuron’s plasma membrane, a process that is regulated by interactions with intracellular trafficking and scaffolding proteins (Heine et al., 2008). Because synaptic transmission occurs within the tightly confined cleft between pre and postsynaptic sites, lateral AMPA receptor diffusion along the plasma membrane can potently alter the number of receptors capable of responding to glutamate release. This modulates postsynaptic sensitivity to transmitter independently of changes in presynaptic function and provides a key mechanism through which neural networks dynamically regulate excitability.

Until recently, AMPA receptor motility in the plasma membrane had only been studied *in vitro*, generally using fluorescence recovery after photobleaching or FRAP. In these experiments, AMPA receptors are labeled with a fluorescent tag that allows receptors to be tracked during live imaging. A segment of plasma membrane containing AMPA receptors is then photobleached through excessive illumination, and researchers measure the time and extent to which the fluorescent signal recovers in the bleached region. A rebound in fluorescence within the bleached membrane segment indicates that AMPA receptors diffused into the region following photobleaching. Estimates of AMPA receptor diffusion using *in vitro* FRAP experiments have been highly variable, ranging from estimates that anywhere between 20% and 100% of AMPA receptors are mobile within the plasma membrane (Chen et al., 2021). Such discrepancies make interpretation of these data challenging and suggest that *in vitro* preparations may alter the biology that exists *in vivo*. Resolving these inconsistencies is of profound importance as it can point to which mechanisms of homeostatic plasticity predominate within the brain.

New results by Chen et al. (2021) in *eNeuro* provide the first *in vivo* estimate of AMPA receptor mobility in the plasma membrane. In experiments with a high degree of technical difficulty, Chen and colleagues used *in utero* electroporation to tag AMPA receptors with fluorescent probes and implanted mice with cranial windows for two-photon *in vivo* imaging once they reached adulthood. They then performed FRAP experiments by photobleaching synaptic spines and measuring the fluorescence recovery in the visual and motor cortices. In both regions, roughly 50% of the fluorescent signal recovered following quenching, suggesting that a significant proportion of AMPA receptors are immobile at basal states in live animals.

Many CNS disorders are characterized by changes in synapse strength and network excitability that could involve alterations in AMPA receptor mobility. Exposure to significant stress, particularly in early life, is one of the strongest risk factors for developing pathologic brain conditions and stress hormones can alter synaptic transmission and plasticity (McEwen et al., 2015). To test the hypothesis that aberrant AMPA receptor diffusion may contribute to stress-induced network dysfunction, Chen et al. (2021) administered corticosterone to one group of mice following baseline FRAP measurements and compared AMPA receptor diffusion to that observed in mice injected with saline. These experiments revealed that AMPA receptor mobility is greatly enhanced following corticosterone injections, suggesting that synapse composition becomes more volatile following periods of stress.

In summary, Chen et al. (2021) demonstrate that synapses *in vivo* exist in a state of intermediate AMPA receptor motility that maximizes the dynamic range over which synapse strength can be bi-directionally modulated. Furthermore, the finding that corticosterone treatment significantly altered these dynamics indicates that systemic factors associated with different physiological states can have immediate and profound impacts on synapse function and network scaling. These rigorous *in vivo* data provide a critical foundation for future *in vivo* and *in vitro* studies of AMPA receptor dynamics aimed at revealing the mechanisms underlying physiological and pathologic synaptic and network function in numerous contexts.

## Synthesis

Reviewing Editor: Christophe Bernard, INSERM & Institut de Neurosciences des Systèmes
